# Influence of intraoperative conventional fluoroscopy versus cone beam CT on long-term clinical outcome in isolated displaced talar fractures

**DOI:** 10.1186/s13018-018-1043-3

**Published:** 2019-01-08

**Authors:** Sven Y. Vetter, Kira Steffen, Benedict Swartman, Marc Schnetzke, Holger Keil, Jochen Franke, Paul Alfred Grützner, Nils Beisemann

**Affiliations:** MINTOS - Medical Imaging and Navigation in Trauma and Orthopaedic Surgery, BG Trauma Center Ludwigshafen, Heidelberg University Hospital, Ludwig-Guttmannstr. 13, 67071 Ludwigshafen, Germany

**Keywords:** Talus fracture, Cone beam CT, Intraoperative imaging, Computed tomography

## Abstract

**Introduction:**

The objective of the study was to compare the radiologic and clinical outcome of patients with an isolated displaced talus fracture treated intra-operatively with either conventional fluoroscopy or additional cone beam computed tomography (CT).

**Methods:**

Conventional intraoperative fluoroscopy was performed in group 1 and cone beam CT was added in group 2. Clinical outcome was assessed using the Foot Function Index (FFI), American Foot and Ankle Society (AOFAS) Ankle-Hindfoot Scale, and the Short-Form 12 (SF-12) survey. In addition, the Kellgren-Lawrence score using X-rays was determined.

**Results:**

Overall, 24 cases were examined (group 1: 8 cases; group 2: 16 cases), with a mean follow up of 6.66 years. The FFI (group 1: 28.85 ± 22.78; group 2: 14.96 ± 15.11 points; *p* = 0.768), the AOFAS (group 1: 69.00 ± 24.71; group 2: 78.79 ± 17.07 points; *p* = 0.438), and the physical and mental component of the SF-12 (group 1: 44.79 ± 12.55; group 2: 47.63 ± 10.69 points; *p* = 0.136) (group 1: 46.19 ± 9.72; group 2: 53.57 ± 8.51; *p* = 0.242) did not differ significantly. Osteoarthritis of the talonavicular, subtalar, and ankle joints assessed using the Kellgren-Lawrence score appeared to be minor in the cone beam CT group but did not show significant differences (*p* = 0.309; *p* = 0.663; *p* = 0.082 respectively).

**Discussion:**

Intraoperative cone beam CT in addition to conventional fluoroscopy might be beneficial in the operative treatment of talar fractures but a statistical significance could not be demonstrated.

## Introduction

Fractures of the talus are rare and constitute only less than 2% of all fractures [1, 2]. In most cases, high-energy trauma with axial stress accounts for these types of fractures but rotational low-energy injuries can also result in avulsions of the talus [3]. Displaced fractures are treated operatively with either closed or open reduction and with a screw, K-Wire, or plate fixation [4]. Due to the retrograde blood supply of the talar body, osteonecrosis or mal-union/nonunion after disruption of the anatomic arterial ring surrounding the talar head occur [5]. An anatomic reduction and a stable fixation of the fracture need to be achieved to lower the rate of complications [6–8]. Intraoperative fluoroscopy to analyze fracture reduction of the talus is implemented in the standard workflow. Despite that, the evaluation of the reconstruction of all joint surfaces and the implant placement can be demanding with conventional fluoroscopy. However, even in complex talar fractures, the intraoperative analysis of the reduction and implant placement is often still evaluated with conventional fluoroscopy [9, 10]. Postoperative computed tomography (CT) is the gold standard to assess the surgical outcome. If necessary, an additional revision surgery is then performed. An intraoperative analysis with a cone beam CT to detect malreduction or insufficient implant placement was found to be advantageous in several anatomical regions [11–13]. Image quality of intraoperative cone beam CT was found to be reasonable for identifying implant placement and fracture reduction of the talus in clinical and cadaveric studies [14–17]. The impact of intraoperative 3D imaging on radiologic and clinical outcome in displaced talar fractures has not been previously published.

The aim of this retrospective cohort analysis was to compare the radiologic and clinical outcome of patients with an isolated displaced talar fracture examined intra-operatively with either conventional fluoroscopy or additional cone beam CT. The hypothesis was that the use of intraoperative cone beam CT leads to an improved reduction and therefore to a superior radiologic and clinical outcome.

## Material and methods

Patients with an isolated displaced fracture of the talus that was operated on between January 2001 and December 2013 were included in the study. Follow-up time was defined to be at least 24 months. The inclusion and exclusion criteria are listed in Table 1.

**Table 1:** Inclusion and exclusion criteria

Inclusion criteria	
Isolated displaced talar fracture	
Operative treatment	
Trauma between January 2001 and December 2013	
Follow-up ≥ 24 months	
Age ≥ 18 years	
Exclusion criteria	
Conservative treatment	
Undisplaced fracture	
Injury of the ipsilateral limb	
Operative treatment with plate, bonegraft or K-Wire	

**Fig. 1:**
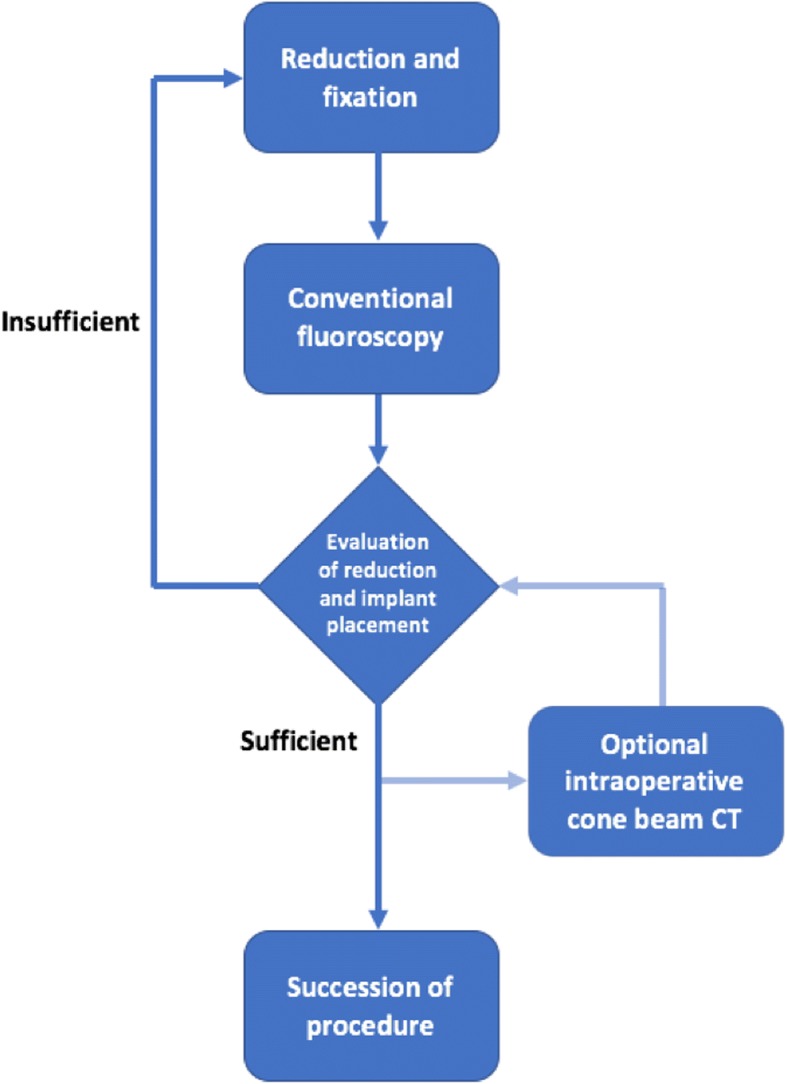
Workflow for the use of intraoperative imaging. The result of reduction and fixation was controlled via conventional fluoroscopy (group 1). After reduction and implant placement, an additional cone beam computed tomography (CT) was performed for group 2. Insufficient reduction or implant placement led to a revision of the reduction and a reevaluation via conventional fluoroscopy and cone beam CT

The operative treatment depended on the morphology of the fracture and the surrounding soft tissue. A soft tissue consolidation was waited for and the surgery was performed when soft tissue swelling had diminished. Severely displaced and closed fractures (grade 3 Oestern and Tscherne) were operated on immediately. All patients were placed in a supine position on a radiolucent carbon fiber table. Either closed or open reduction was necessary to achieve an anatomic reduction. The osteosynthesis in all cases was performed with 4.5 mm cannulated screws (DepuySynthes, Johnson&Johnson, USA). K-wires and plates as well as bone grafting were not used in this study. After reduction, conventional intraoperative fluoroscopy was performed in group 1 and an additional cone beam CT was performed in group 2. The use of intraoperative cone beam CT depended on the surgeon’s assessment. A randomization between the groups was not performed. The cone beam CT scan was performed with the SIREMOBILE Iso-C3D (Siemens Healthcare, Forchheim, Germany) and from March 2005 onwards with the ARCADIS Orbic 3D (Siemens). With these motorized, 3D C-arms, 100 serial fluoroscopic images were obtained during a 190° orbital rotation. From these images, a 3D dataset was obtained with an edge length of 120 mm. The scan typically lasted 1 min (ARCADIS Orbic 3D scanner) or 2 min (SIREMOBILE Iso-C3D scanner). Multiplanar reconstructions in the three standard planes (axial, semi-coronal, and sagittal) were then created to assess the reduction quality and implant position. Including the analysis of the images and decision-making, an intraoperative cone beam CT scan lasted approximately 5 min. If reduction or implant placement was insufficient, a correction was carried out. The intraoperative workflow is illustrated in Fig. 1. Postoperative X-ray images were obtained to analyze fracture reduction. All patients were immobilized in a non-weight-bearing orthopedic boot in neutral position for a period of 8 weeks with early foot and ankle motion followed by progressive weight-bearing for a period of 6 weeks. Anteroposterior, lateral, and mortise radiographs of the ankle joint were routinely made at 2 days, 6 weeks, and 12 weeks post-operatively to evaluate fracture reduction according to the criteria proposed by Lindvall et al. [18]. Additional radiography, CT scans, and magnetic resonance imaging were performed if necessary. For the final follow-up, anteroposterior, lateral, and mortise radiographs were obtained. The clinical outcome was determined with a clinical investigation (range of motion, ROM) of the ankle joint, Foot Function Index, American Foot and Ankle Society (AOFAS) Ankle-Hindfoot Scale, and the Short-Form 12 survey. To classify the severity of osteoarthritis of the upper ankle joint, the Kellgren-Lawrence score was applied on standard X-ray images during the follow-up at least 2 years after surgery. The patients were allocated in two groups: conventional intraoperative fluoroscopy (group 1) versus additional cone beam CT (group 2).

The statistical analysis was performed using SPSS (Version 21.0.0.2, IBM, Armonk, New York, USA) and Microsoft Excel 2013 (Version 15.0.4779.1001, Microsoft, Redmond, USA). Patients from the two groups were compared based on the aforementioned parameters. The statistical significance level was set to *p* < 0.05. For ordinal and nominal type variables, a Chi-squared test or Fisher’s exact test was applied. Interval type variables were compared using the *t* test if distributed normally. The Mann-Whitney *U* test was used for other distributions. The influence of covariates was determined with an analysis of covariance (ANCOVA).

All procedures performed in the study involving human participants were in accordance with the ethical standards and was approved by the state ethics committee.

## Results

In total, 32 patients with an isolated talus fracture underwent reduction and screw fixation during August 2001 and December 2013. Twenty-four patients were included in this study (follow-up rate 75%). The demographic data, mechanism of injury, and fracture classification according to Marti-Weber are detailed in Table 2.

**Table 2:** Demographic data and distributions of mechanism of injury and fracture classification

	Conventional fluoroscopy	Cone beam CT
Age		
Average	34.1 ± 11.1 years	41.8 ± 14.3 years
Sex		
Male	62.5% (5)	50.0% (8)
Female	37.5% (3)	50.0% (8)
Side		
Left	50.0% (4)	62.5% (10)
Right	50.0% (4)	37.5% (6)
Fracture classification Marti/Weber		
III	62.5% (5)	87.5% (14)
IV	37.5% (3)	12.5% (2)

**Fig. 2:**
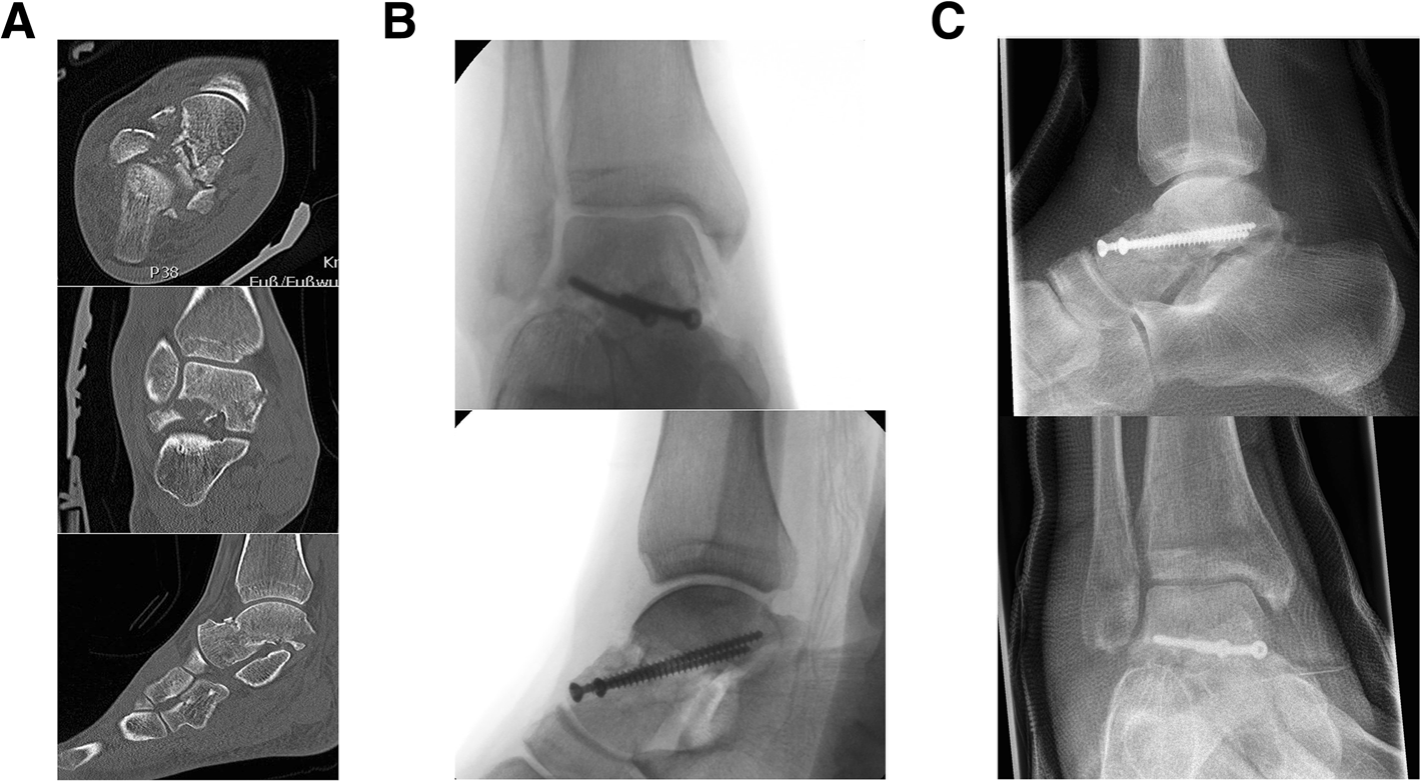
**a** Group 1 preoperative CT. **b** Group 1 intraoperative fluroscopy. **c** Group 1 postoperative X-ray

**Fig. 3:**
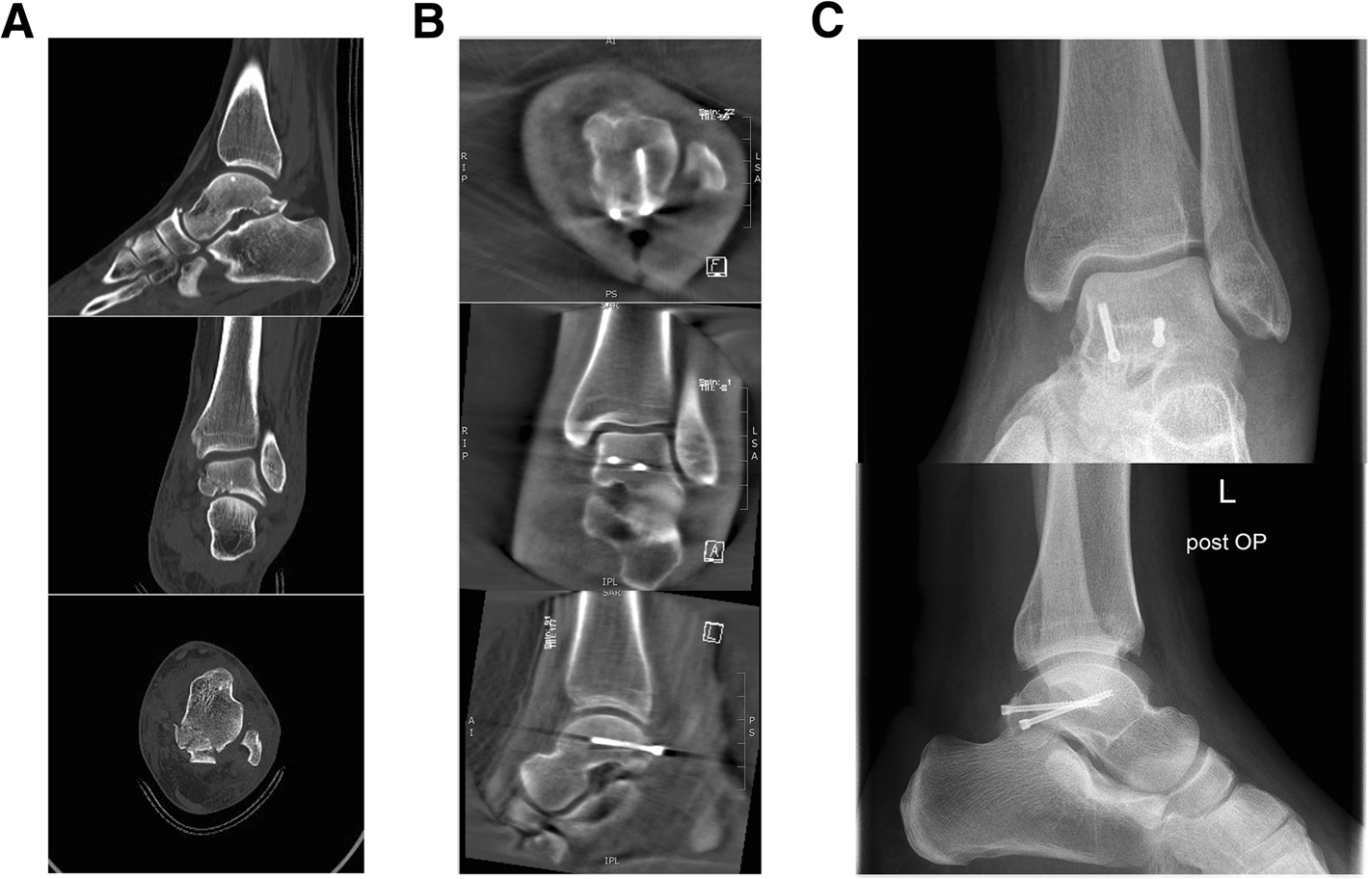
**a** Group 2 preoperative CT. **b** Group 2 intraoperative cone beam CT. **c** Group 2 postoperative X-ray

Pre-, intra-, and postoperative images are listed as Figs. 2 and 3.

The mean follow-up time averaged 6.66 ± 4.16 years (range 2.08–14.58 years). In 16 cases (66.7%), an intraoperative cone beam CT was performed to assess fracture reduction and implant placement. Table 3 lists the method of operative treatment and reasons for intraoperative revision based on the cone beam CT scan results.

**Table 3:** Consequences after utilization of intraoperative cone beam computed tomography

Consequence	Occurrence	Percentage
No consequence	11	68.8
Correction of screw placement	4	25.0
Correction of reduction	1	6.2

**Fig. 4:**
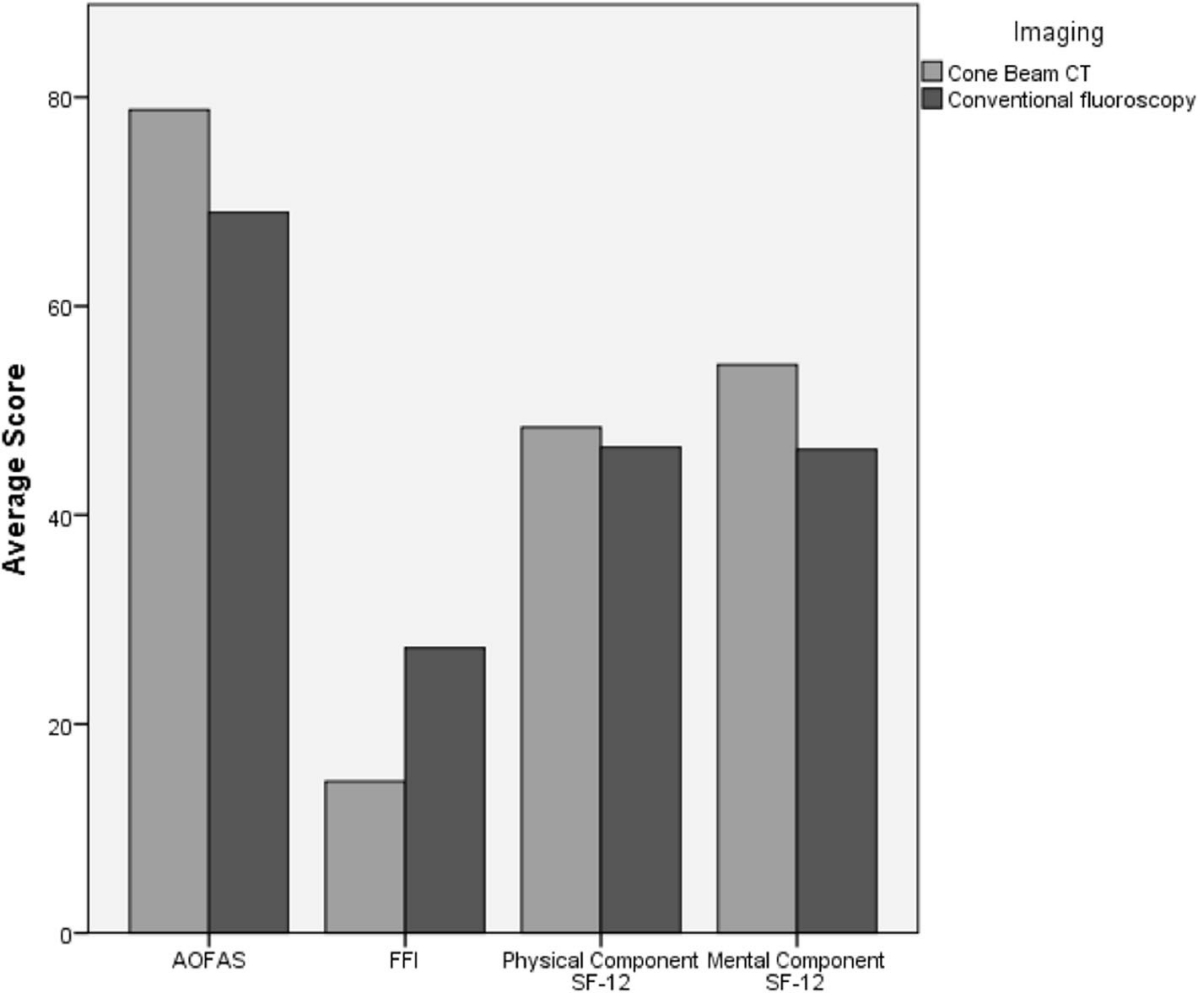
Comparison of the average scores depending on intraoperative imaging (cone beam computerized tomography or conventional fluoroscopy). The specified scores are the total score of the Ankle-Hindfoot Scale of the American Orthopedic Foot and Ankle Society (AOFAS), the Foot Function Index (FFI), and the Physical and Mental Component Score of the Short Function 12 (SF-12)

**Fig. 5:**
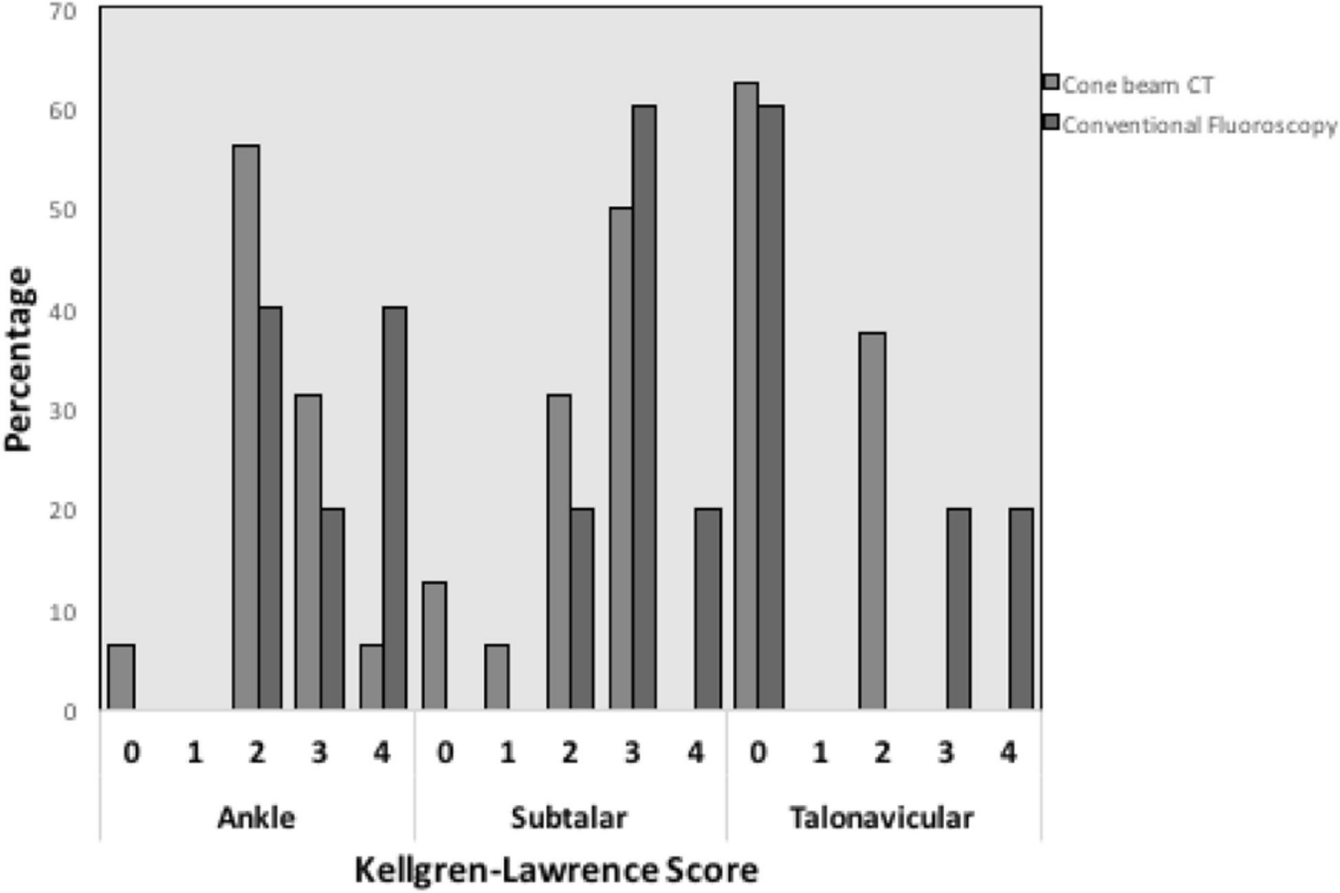
Occurrence of osteoarthritis in talar joints depending on intraoperative imaging. Displayed is the percentage of patients in the respective group presenting each Kellgren-Lawrence grade (cone beam computerized tomography vs. conventional fluoroscopy). The distribution is shown for each of the joints adjacent to the talus: the ankle, subtalar, and talonavicular joint

The operation time did not differ significantly between the groups (group 1 80.0 ± 41.3 min; group 2 72.9 ± 37.7 min; *p* = 0.692). The ROM did not reveal significant differences between the groups (group 1: 37.9 ± 21.8°; group 2 44.6 ± 18.0; *p* = 0.457). The physical and mental component score SF-12, as well as the AOFAS Ankle-Hindfoot Scale, and the Foot Function Index score differed but not significantly. The results are displayed in Fig. 4. Osteoarthritis of the talonavicular, subtalar, and ankle joints appeared to be minor in the cone beam CT group but did not show significant differences (Fig. 5).

## Discussion

The objective of the study was to analyze the radiologic and clinical outcome of patients with an isolated displaced talar fracture treated with either conventional fluoroscopy or additional cone beam CT intra-operatively. The hypothesis was that patients treated with intraoperative cone beam CT have a significant superior radiologic and clinical outcome. A tendency of the data toward the cone beam CT group is evident but a significant improvement of the clinical and radiological outcome cannot be conveyed from the results. Therefore, the hypothesis of the study is refused.

Intraoperative cone beam CT showed a beneficial impact on the clinical outcome of patients with calcaneal fractures [12]. Seventy-seven patients were included in the study by Franke et al. detecting statistically significant differences in the American Foot and Ankle Society (AOFAS) Ankle-Hindfoot Scale between the groups.

The clinical outcome in the study was assessed with a clinical investigation (ROM), Foot Function Index, AOFAS Ankle-Hindfoot Scale, and the Short-Form 12 survey. The ROM did not reveal significant differences between the two groups and neither did the clinical surveys. However, the cone beam CT group did tend to exhibit superior results in ROM and all clinical tests. The score of the conventional fluoroscopy group (average 69.0 ± 24.7) in the AOFAS is comparable to publicized data by Sanders et al. (71 ± 19 points), Ohl et al. (67 points), and Liu et al. (72.8 ± 17.3 points) [9, 19, 20]. The cone beam CT group scored higher with 78.8 points. The clinical outcome of the patients lacks good and excellent results due to the exclusion of non-displaced talar fractures in this study, which generally have a superior prognosis [21].

Osteoarthritis of the ankle, subtalar, and talonavicular joints was detectable in almost all patients at least 2 years after trauma. Kellgren-Lawrence score grade 4 appeared to occur more frequently in the conventional fluoroscopy group with 33% in the subtalar/talonavicular joint and 50% in the ankle joint respectively. A strong tendency was evident (*p* = 0.082), but a significant difference to the cone beam CT group could not be detected. Several studies reveal a high rate of posttraumatic osteoarthritis in the adjacent joints after displaced talar fractures but omitted differentiating the grade according to the Kellgren-Lawrence score [9, 19, 20, 22, 23].

The rate of an osteonecrosis did not differ significantly between groups but revealed a high percentage of 57.9%. Similar results could be observed by Lindvall et al. and Hawkins [18, 24]. Cebesoy et al. proposed that fractures reduction might not influence the risk of avascular necrosis, especially after Hawkins type 2 and 3 fractures [25]. Lindvall et al. consented to this view that in particular the severity of the injury itself, and not the fracture fixation, may be responsible for higher rates of osteonecrosis [18]. Irrespective of this, intra-articular implant placement or insufficient reduction with steps and gaps of the joint surface are not favorable for the clinical outcome.

As the isolated fracture of the talus is a rare entity, since talar fractures are often accompanied by other injuries, the total number of patients considered in the study is small. This corresponds to the data published by Sanders et al. in a retrospective cohort study [20]. Another limitation of the study is the follow-up rate of only 75%, although this is comparable with other studies [6, 18, 20, 22]. This low rate is partly due to the rare occurrence of the fracture and the consecutive long follow-up times, which makes contacting the patients more difficult. Furthermore, the usage of cone beam CT to analyze reduction and implant placement was not randomized but relied on the intraoperative surgeon’s assessment.

In this study, only closed fractures were examined. This can be explained by the inclusion criteria of an isolated fracture. Severe soft issue impairment is often accompanied with multiple injuries of the limb.

Due to intraoperative imaging, intra-articular implant placement could be excluded in the cone beam CT group. Efforts were made to avoid steps and gaps in the articular surface but sometimes these could not be eliminated due to fracture morphology.

## Conclusion

The surgical treatment of displaced talar fractures continues to be challenging. Intraoperative cone beam CT additional to conventional fluoroscopy might be beneficial for the surgeon in the operative treatment even though the results of the study do not reveal a significant improvement of the clinical and radiologic outcome in the cone beam CT group. A larger cohort of this rare injury appears to be necessary to reveal statistical significant differences.
